# Exploring the molecular mechanisms of ferroptosis-related genes in periodontitis: a multi-dataset analysis

**DOI:** 10.1186/s12903-024-04342-2

**Published:** 2024-05-27

**Authors:** Jili Chen, Lijia Ou, Weizhen Liu, Feng Gao

**Affiliations:** 1 Department of Periodontics, Panyu Branch, Stomatological Hospital, School of Stomatology, Southern Medical University, No.366 Jiangnan Dadao Nan, Haizhu District, Guangzhou, Guangdong 510220 China; 2 Department of Histology and Embryology, Xiangya School of Medicine, Central South University, No. 172 Tongzipo Road, Yuelu District, Changsha, 410006 China

**Keywords:** Bioinformatic analysis, Multiomic datasets, Periodontitis, Ferroptosis, Immunoinfiltration

## Abstract

**Purpose:**

This study aims to elucidate the biological functions of ferroptosis-related genes in periodontitis, along with their correlation to tumor microenvironment (TME) features such as immune infiltration. It aims to provide potential diagnostic markers of ferroptosis for clinical management of periodontitis.

**Methods:**

Utilizing the periodontitis-related microarray dataset GSE16134 from the Gene Expression Omnibus (GEO) and a set of 528 ferroptosis-related genes identified in prior studies, this research unveils differentially expressed ferroptosis-related genes in periodontitis. Subsequently, a protein–protein interaction network was constructed. Subtyping of periodontitis was explored, followed by validation through immune cell infiltration and gene set enrichment analyses. Two algorithms, randomForest and SVM(Support Vector Machine), were employed to reveal potential ferroptosis diagnostic markers for periodontitis. The diagnostic efficacy, immune correlation, and potential transcriptional regulatory networks of these markers were further assessed. Finally, potential targeted drugs for differentially expressed ferroptosis markers in periodontitis were predicted.

**Results:**

A total of 36 ferroptosis-related genes (30 upregulated, 6 downregulated) were identified from 829 differentially expressed genes between 9 periodontitis samples and the control group. Subsequent machine learning algorithm screening highlighted 4 key genes: SLC1A5(Solute Carrier Family 1 Member 5), SLC2A14(Solute Carrier Family 1 Member 14), LURAP1L(Leucine Rich Adaptor Protein 1 Like), and HERPUD1(Homocysteine Inducible ER Protein With Ubiquitin Like Domain 1). Exploration of these 4 key genes, supported by time-correlated ROC analysis, demonstrated reliability, while immune infiltration results indicated a strong correlation between key genes and immune factors. Furthermore, Gene Set Enrichment Analysis (GSEA) was conducted for the four key genes, revealing enrichment in GO/KEGG pathways that have a significant impact on periodontitis. Finally, the study predicted potential transcriptional regulatory networks and targeted drugs associated with these key genes in periodontitis.

**Conclusions:**

The ferroptosis-related genes identified in this study, including SLC1A5, SLC2A14, LURAP1L, and HERPUD1, may serve as novel diagnostic and therapeutic targets for periodontitis. They are likely involved in the occurrence and development of periodontitis through mechanisms such as immune infiltration, cellular metabolism, and inflammatory chemotaxis, potentially linking the ferroptosis pathway to the progression of periodontitis. Targeted drugs such as flurofamide, L-733060, memantine, tetrabenazine, and WAY-213613 hold promise for potential therapeutic interventions in periodontitis associated with these ferroptosis-related genes.

**Supplementary Information:**

The online version contains supplementary material available at 10.1186/s12903-024-04342-2.

## Introduction

Periodontal diseases are regarded as the most common diseases of mankind. The prevalence rate of periodontal disease assumes a clear growth tendency [1], Globally, the severe form of the disease has a prevalence of 11%, affecting 743 million individuals [2, 3]. Periodontitis is a chronic multifactorial inflammatory disease associated with the accumulation of dental plaque (which will be referred to as dental biofilm/biofilm), and characterised by progressive destruction of the teeth-supporting apparatus, including the periodontal ligament and alveolar bone [4]. In advanced cases, periodontitis leads to a substantially negative effect on oral health related quality of life (OHRQoL),while successful management may improve patients’ OHRQoL.The previous study has reported about the intricate molecular mechanism underlying this periodontitis [5]. However, the role of specific genes, cells, or cellular mechanisms involved in the pathogenesis of periodontitis are still unclear, and there are no pre-diagnostic markers or therapeutic targets available for such inflammatory lesions [6].

The investigation of molecular mechanism and diagnostic marker at the cellular or molecular level are important for periodontitis [7]. For example,R Ientile et al. observed that transglutaminase gene expression may be modified in response to chronic injury and play a key role in gingival remodelling/healing and adaptive processes [8]; Gaetano Isola et al. indicated that gingival crevicular fluid miRNAs is closed associated with the risk of periodontitis [9]; As is widely known that ferroptosis as an iron-dependent form of non-apoptotic cell death was descripted in 2012,and it was defined as the oxidative response of the intracellular microenvironment regulated by glutathione peroxidase 4 (GPX4), a form of regulatory cell death triggered by stimulation that can be inhibited by iron chelators and lipophilic antioxidants in 2018 [10]. Recently,there has been mounting interest in the function and role of ferroptosis in various diseases [11, 12]. Interestingly, several studies have confirmed that ferroptosis is indeed closely related to the inflammatory response including the bacterial inflammatory reaction in the oral cavity such as periodontitis [13, 14]. Wang et al.explored the relationship between antioxidant system and periodontitis,and considered ROS plays a key role in periodontitis due to excessive lipid peroxidation and tissue damage [15–17]; In Meuric’s study,periodontitis is associated between serum iron biomarkers in genetic haemochromatosis patients,they aslo demonstrate that increased transferrin saturation is associated with oral dysbiosis characterized by elevated proportions of periopathogens, which may participate in periodontitis severity [18]; Furthermore,Bains et al.found that periodontitis patients have the lower concentrations of reduced glutathione (GSH) in serum and gingival crevicular fluid, and periodontal therapy restores the redox balance [19, 20]. Based on the compelling evidence regarding the correlation between periodontitis and ferroptosis three elements (ROS,GSH and Iron) presented in the aforementioned studies, we reasonably hypothesize a necessary link between ferroptosis and the pathogenesis of periodontitis. Furthermore, we aim to explore the role of ferroptosis-related genes in clinical patients with periodontitis by utilizing bioinformatics techniques across multiple datasets.

In this study, we identified differentially expressed genes by analyzing a periodontitis-related microarray dataset from the Gene Expression Omnibus (GEO) database, alongside 528 ferroptosis-associated genes identified in prior research. This analysis was followed by the construction of a protein–protein interaction network. Subsequently, we investigated the subtypes of periodontitis, corroborating our findings through assessments of immune cell infiltration and gene set enrichment. To discern potential diagnostic markers of ferroptosis in periodontitis, we employed two machine learning algorithms. The diagnostic efficacy, potential therapeutic targets, and immune associations of these markers were then rigorously evaluated. Lastly, we constructed a microRNA (miRNA)-gene interaction network to further elucidate the molecular mechanisms at play.This finding could pave the way for an advanced comprehension of ferroptosis's role in the pathogenesis of periodontitis, thereby laying a theoretical foundation for subsequent investigations into the molecular mechanisms underlying the disease.

## Materials

### Data download

The GEO database (https://www.ncbi.nlm.nih.gov/geo/info/datasets.html), known as GENE EXPRESSION OMNIBUS, is a gene expression database created and maintained by the National Center for Biotechnology Information (NCBI) in the United States. The search in the NCBI GEO public database was conducted using keywords "periodontitis," "single-cell," "gingiva," and "transcriptome," with the species set to "Homo sapiens," resulting in the inclusion of GSE171213 and GSE16134. GSE171213 is a single-cell dataset comprising nine samples, with five samples from diseased subjects and four from control subjects. GSE16134 is a transcriptome dataset comprising 310 samples, with the annotation file labeled as GPL570, including 241 samples from patients with the disease and 69 samples from healthy individuals. The ferroptosis gene set utilized in this analysis consists of 564 genes associated with ferroptosis.

### GO and KEGG function notes

The functional annotation of crucial gene sets was conducted using the Metascape database (www.metascape.org), aiming to comprehensively explore the functional relevance of these genes. Gene Ontology (GO) analysis and Kyoto Encyclopedia of Genes and Genomes (KEGG) pathway analysis were performed for specific genes. Statistical significance was considered when Min overlap was ≥ 3 and *p* ≤ 0.01.

### Screening of key genes

Firstly, differential gene analysis was employed to identify significant variances within the GEO dataset (adj.*P*.Val < 0.05 and |logFC|> 0.585). Subsequently, two distinct algorithms, RandomForest and SVM, were applied to refine the candidate gene set further. Among them, randomForest is an integrated learning algorithm based on a decision tree, which selects multiple samples from the sample set as a training set by the method of sampling back and generates a decision tree from the sample set obtained by sampling.

At each generated node, features are randomly and non-repetitively selected. Utilizing these features, the sample set is partitioned, identifying the optimal feature for partitioning and determining the predicted outcome. This study assesses the importance of features using the random forest algorithm, constructing a total of 1000 classification trees with 50 iterations. The evaluation of feature importance is based on %IncMSE (% Increase in Mean Squared Error).S VM-RFE is a machine learning method based on Support Vector Machines (SVM). It identifies optimal variables by eliminating feature vectors generated by SVM. The Support Vector Machine model is established using the "e1071" package, facilitating the further recognition of these biomarkers' diagnostic value for the disease. Ultimately, the top 15 features are retained for subsequent analysis.

### Development and verification of artificial neural network model

A neural network model was developed utilizing the R package "neuralnet" based on identifying key genes using machine learning. First, transform the expression levels of key genes into a "gene score" based on their respective expression levels. For a specific sample, the expression level of a particular gene is compared to the median expression level across all samples. Subsequently, the hidden layer of the Artificial Neural Network (ANN) is configured as 5, and the gene weights are computed through the "gene score." Finally, an ANN diagnostic model is established, and its diagnostic performance is evaluated through the Area Under the Curve (AUC).

### Single-cell analysis

First, the expression profile was imported using the Seurat package, and low-expressed genes were filtered out (nFeature_RNA > 300 & percent. mt < 30). Subsequently, the data underwent standardization, normalization, and PCA analysis. The optimal number of principal components (20) was determined by observing the ElbowPlot. The t-SNE analysis was then employed to reveal the spatial relationships between clusters. The clusters were annotated using the annotation file, HumanPrimaryCellAtlasData, provided by the celldex package. Each cluster was individually annotated to cells with significant relevance to disease occurrence.

### Analysis of immune cell infiltration

The CIBERSORT algorithm was employed to analyze RNA-seq data of periodontitis patients from different subgroups. This analysis aimed to infer the relative proportions of 22 immune infiltrating cell types. Subsequently, Pearson correlation analyses were conducted to assess the correlation between risk scores and the content of immune cells. A significance level of *P* < 0.05 was considered statistically different.

### GSEA enrichment analysis

Performing Gene Set Enrichment Analysis (GSEA; http://www.broadinstitute.org/gsea) on the expression spectrum of periodontitis patients to identify differentially expressed genes between the high-expression and low-expression groups. Utilizing maximum and minimum gene set sizes of 500 and 15 genes, respectively, we filtered the gene sets. Following 100 permutations, we obtained enriched gene sets based on a significance threshold of *P* < 0.05 and a false discovery rate (FDR) of 0.25.

### Drug targeting prediction analysis

The Connectivity Map (CMap) is a gene expression profile database developed by the Broad Institute based on interventions in gene expression. It is primarily utilized to elucidate functional connections between small-molecule compounds, genes, and disease states. The database encompasses gene expression data before and after the treatment with 1309 small-molecule drugs across five human tumor cell lines. The treatment conditions are diverse, involving various drugs, concentrations, and treatment durations, among other factors. In this study, differential gene expression in periodontitis is employed to predict targeted therapeutic drugs for periodontitis.

### Statistical analysis

All statistical analyses were conducted using R language (version 4.1). All statistical tests were two-sided, with *p* < 0.05 considered statistically significant. We used Pearson correlation coefficients to assess the relationships between different variables. Specifically, we calculated the correlation coefficients and their associated *p*-values. For comparisons between two groups, we employed the Wilcoxon test to examine whether there were significant differences in sample means between different groups.

## Results

### Identifying differentially expressed candidate genes associated with ferroptosis in periodontitis

**Fig. 1:**
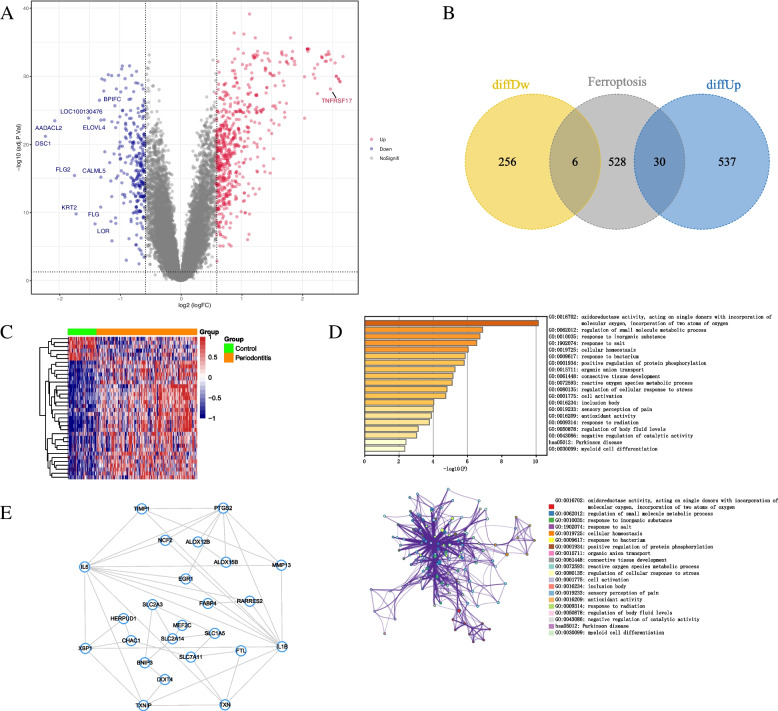
Screening of the differentially expressed ferroptosis related genes in periodontitis. **A** The volcano map of GEO dataset GSE16134. Upregulated genes are marked in red; downregulated genes are marked in blue; Not significant different genes are marked grey. **B** Venn diagram shows the overlap of genes between differentially expressed genes in GSE16134 and ferroptosis related genes. **C** Clustered heatmap of differentially expressed ferroptosis related genes. **D** KEGG enrichment analysis of differentially expressed ferroptosis related genes in GSE16134. **E** The PPI network constructed based on differentially expressed ferroptosis related genes in current study: the line represented interaction; the node represented different differentially expressed ferroptosis related genes

We conducted a differential gene analysis on the GEO dataset GSE16134 using the limma package, comparing control samples with periodontitis samples. The criteria for selecting differentially expressed genes were set as adj.P.Val < 0.05 and |logFC|> 0.585. Ultimately, we identified 567 upregulated genes and 262 downregulated genes (Fig. 1A). This includes 36 ferroptosis-related genes (30 upregulated genes; 6 downregulated genes), as illustrated in Fig. 1B-C. Ultimately, we consider these 36 differentially expressed genes related to ferroptosis as a candidate gene set for further analysis. Through pathway analysis using the Metascape database, it was revealed that these candidate genes are primarily enriched in pathways such as regulation of small molecule metabolic process, response to inorganic substances, and response to salt (Fig. 1D). Simultaneously, we conducted protein-protein interaction network analysis on these genes using Cytoscape software (Fig. 1E).

### Identifying key genes associated with ferroptosis in periodontitis

**Fig. 2:**
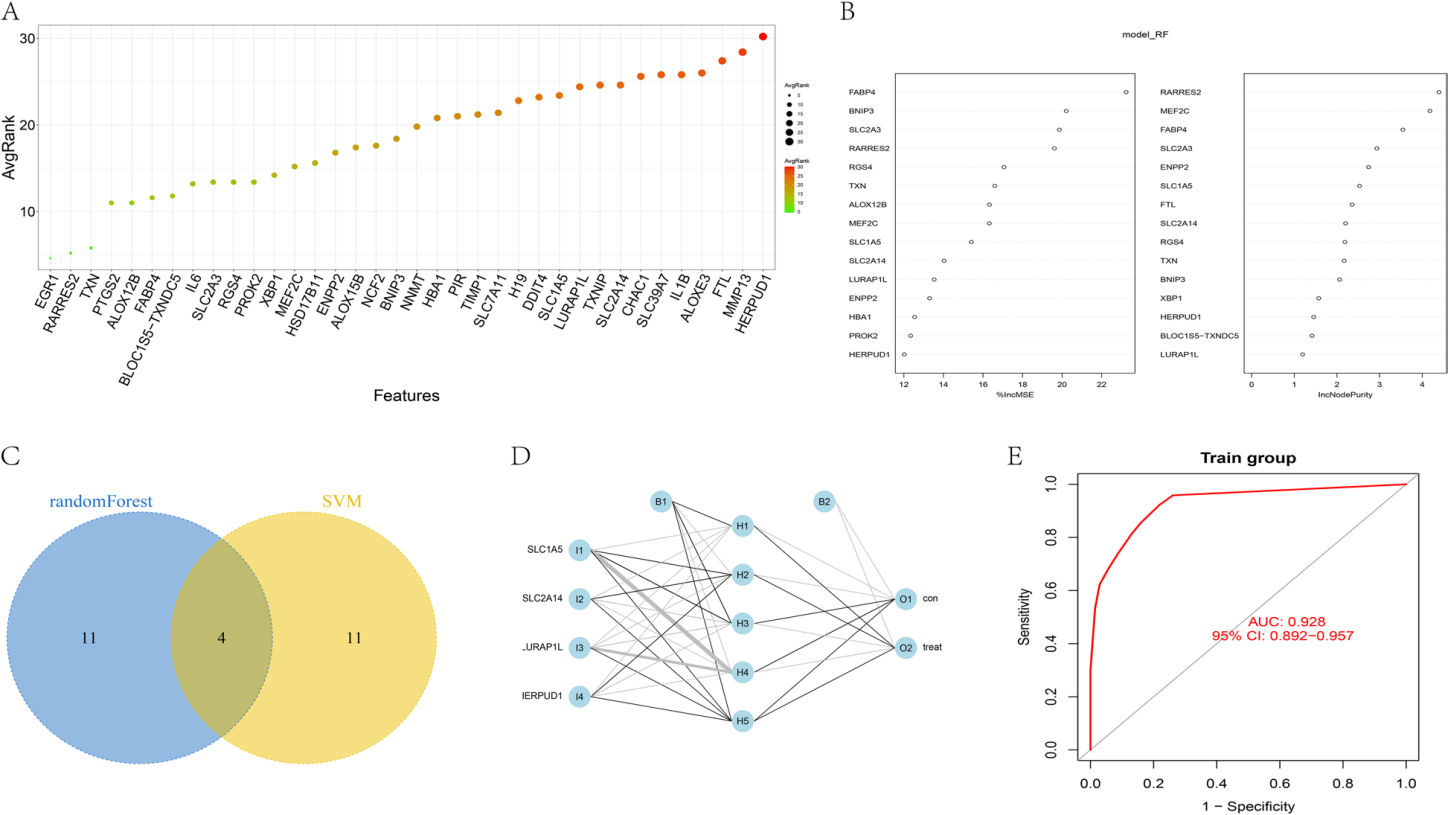
Screening of key genes. **A** Variable importance in the random forest models based on the above 36 candidate genes for the prediction of the role of disease perturbations. **B** The Top 15 genes revealed by SVM analyses according to Mean Decrease Accuracy (left) and Mean Decrease Gini (right). **C** The VENN diagram revealed the overlap of 4 differentially expressed ferroptosis related genes in current study. **D** Network structure diagram for 4 differentially expressed ferroptosis related genes. **E** The result of receiver operating characteristic (ROC) curve analysis for above 4 genes: X-axis represented specificity, Y-axis represented sensitivity

In order to further screen key genes associated with disease perturbations, we conducted random Forest and SVM analyses on the aforementioned 36 candidate genes (Fig. 2A-B). Among them, four genes consistently ranked among the Top 15 feature genes in both rounds of feature selection (Fig. 2C). These genes are SLC1A5, SLC2A14, LURAP1L, and HERPUD1. Utilizing the "neuralnet" R package, we constructed an artificial neural network model based on the GSE16134 dataset. The expression of key genes, identified through machine learning, was transformed into a "gene score" marked as 0/1. We computed the weights of all genes to best discriminate between normal and diseased samples. Subsequently, a diagnostic artificial neural network model was established based on these gene weights (Fig. 2D). The performance of this model, as indicated by the AUC in the dataset, was 0.928 (Fig. 2E), suggesting its robust predictive capability.

### scRNA analysis in periodontitis

**Fig. 3:**
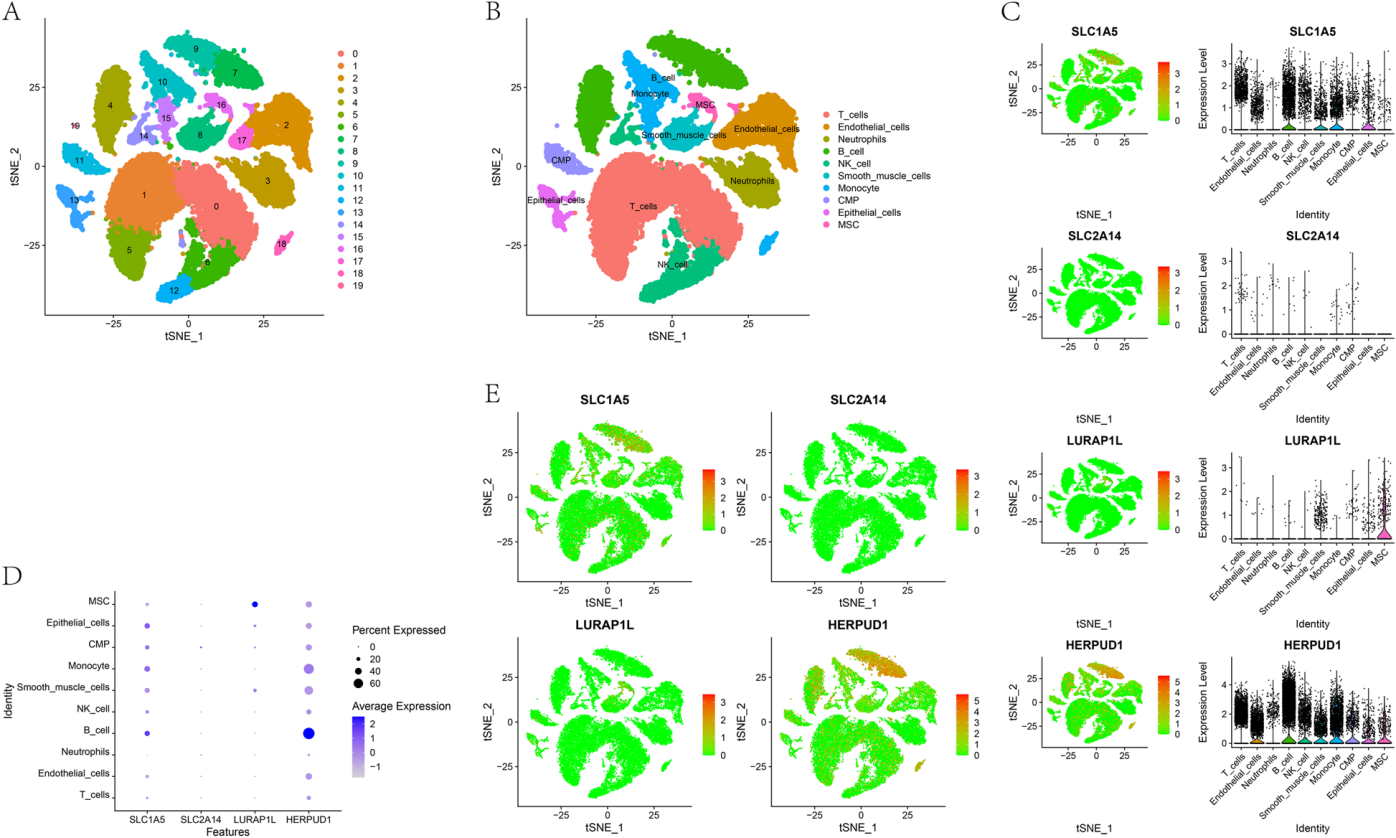
scRNA analysis in periodontitis. **A** The cluster analysis performthe t-distributed stochastic neighbor embedding (t-SNE) was employed for cluster visualization. Each distinct color in the plot corresponds to a cell cluster identified after clustering. Each data point in the scatter plot represents an individual cell, and the numerical labels in the figure indicate the cluster identifiers for each group. The graphical representation indicates a total of 20 distinct cell clusters identified from the clustering process. **B** The t-SNE analysis reveals the categorization of 20 clusters in distinct immune cell types. **C**-**E** The expression of SLC1A5, SLC2A14, LURAP1L, and HERPUD1 across various subtypes of immune cells

In this analysis, we utilized expression profiles from nine periodontitis-associated tissue samples. Employing the FindClusters algorithm, we identified 20 subtypes, as illustrated in Fig. 3A. We annotate each subtype through the R package "SingleR," and 20 clusters are annotated into 10 B cell categories: T cells, Endothelial cells, Neutrophils, B cells, NK cells, Smooth muscle cells, Monocyte, CMP, Epithelial cells and MSC (Fig. 3B). The expression of four key genes in cellular subtypes is illustrated in the figure (Fig. 3C-E).

### Correlation analysis between four key genes and immune microenvironment characteristic factors

**Fig. 4:**
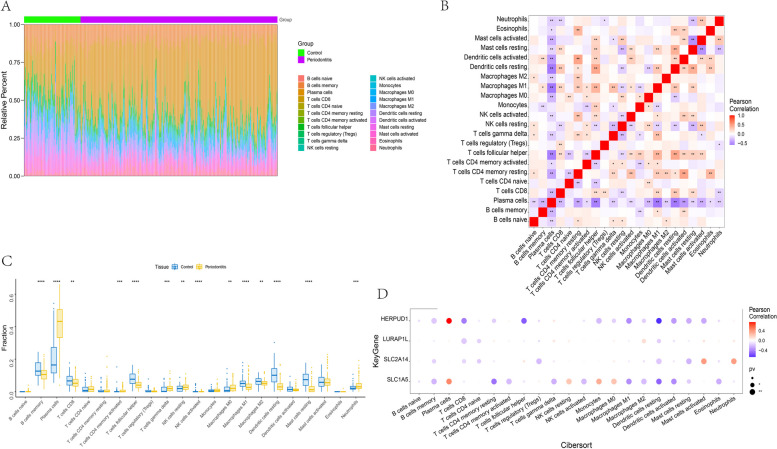
Correlation analysis between four key genes and immune cells. **A** Expression profiling of immune-infiltrating cells in periodontitis patients was conducted using heat map analysis; **B** Pearson correlation analysis was employed to assess the expression correlation among immune-infiltrating cells in periodontitis patients; **C** Comparison of the expression profiles of immune-infiltrating cells between inflammatory tissues of periodontitis patients and the control group; **D** Comparative analysis of the correlation between the expression of four key genes and immune-infiltrating cells

**Fig. 5:**
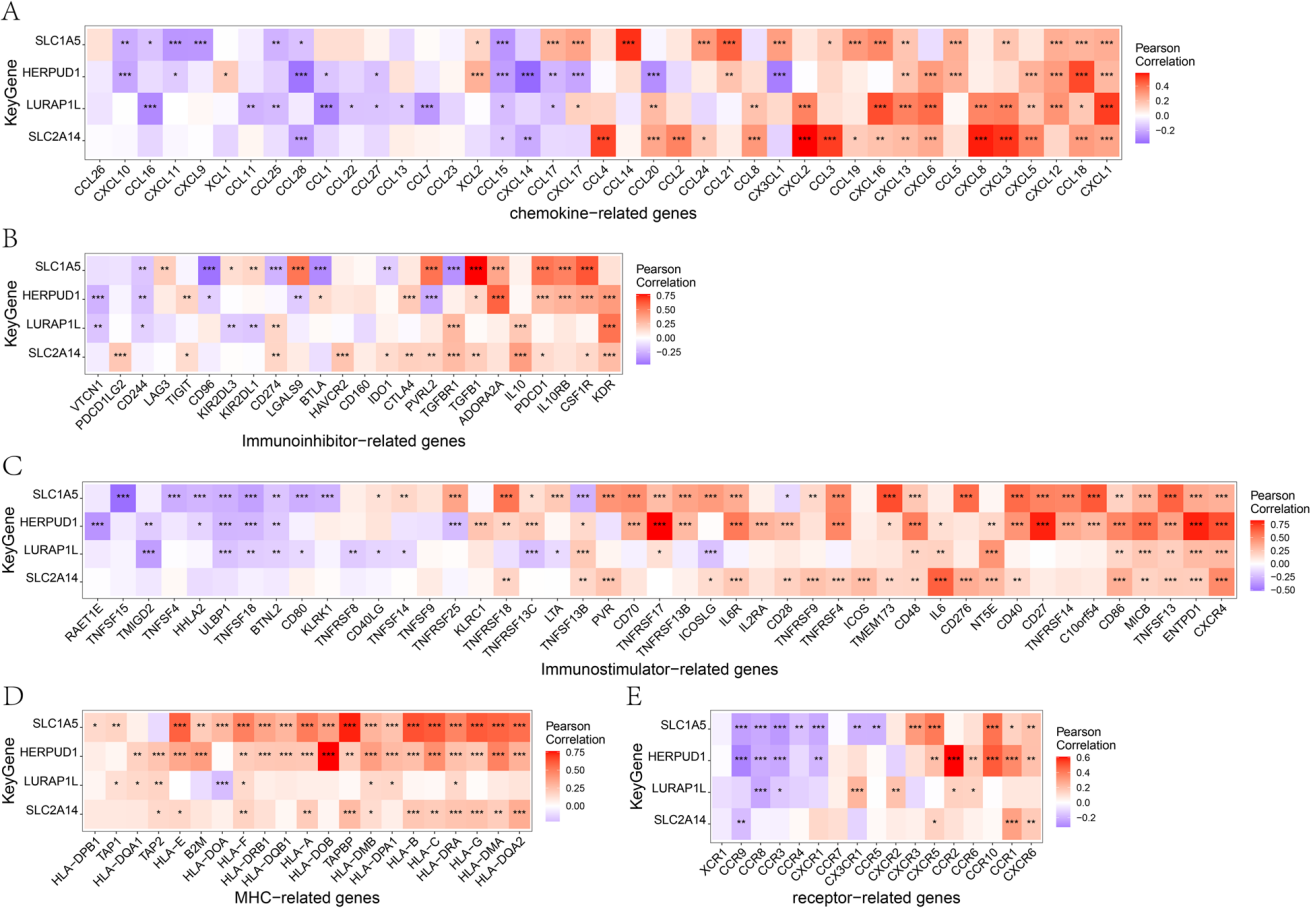
Heatmap between four key genes and immune microenvironment characteristic factors. Heatmap shows the relationship between key genes and chemokine-relative genes(**A**)/immunoinhibitor-relative genes(**B**)/immunostimulator-relative genes(**C**)/MHC-relative genes(**D**)/receptor-relative genes(**E**). Red indicates a positive correlation, purple indicates a negative correlation. The number of asterisk designates the correlation coefficient between key genes and TME characteristic factors

The microenvironment is primarily composed of immune cells, extracellular matrix, various growth factors, inflammatory factors, and specific physicochemical features. It significantly influences the diagnosis and clinical treatment sensitivity of diseases. By analyzing the relationship between key genes and immune infiltration in the periodontitis dataset, we further explore the potential molecular mechanisms through which these key genes influence the progression of periodontitis. The research findings indicate that the proportion of immune cell content in each patient and the correlations between immune cells are illustrated in the figures (Fig. 4A-B). Furthermore, compared to patients with periodontitis, the control group samples showed significantly elevated levels of B cells memory and T cells CD8, while the levels of Plasma cells and T cells gamma delta were significantly decreased (Fig. 4C). We further investigated the relationship between key genes and immune cells, revealing a strong correlation between these key genes and multiple levels of immune cell infiltration. The expression levels of the genes HERPUD1 and SLC1A5 were significantly positively correlated with the infiltration level of Plasma cells. In contrast, there was a significant negative correlation between the infiltration levels of B cell memory and Macrophages M1. The expression levels of genes LURAP1L and SLC2A14 show a significant negative correlation with the infiltration levels of T cells CD8 and T cells CD4 naive (Fig. 4D). We further obtained the correlation between these key genes and various immune factors from the TISIDB database, including immune modulators, chemokines, etc. The results revealed a strong correlation between the key genes and immune factors such as chemokine-related genes, immuoihibitor related genes, immuostimulator related genes, MHC-related genes and receptor related genes (Fig. 5A-E). These analyses confirmed the close association of these key genes with the level of immune cell infiltration, indicating their significant roles in the immune microenvironment.

### Pearson’s correlation analysis between four key genes and pathogenic genes associated with periodontitis

**Fig. 6:**
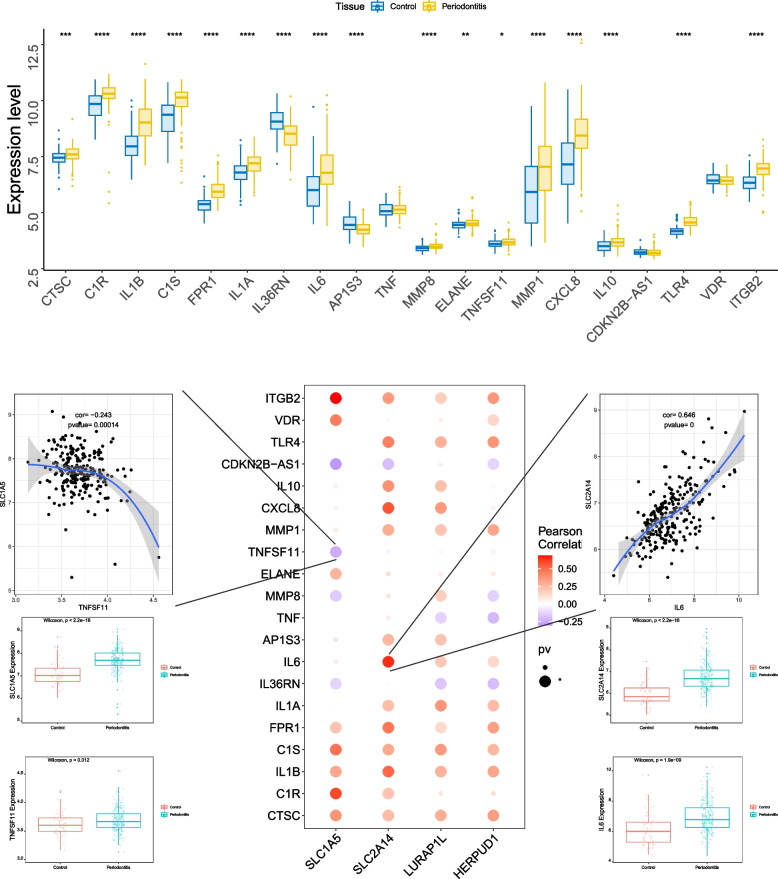
Pearson’s correlation analysis between four key genes and pathogenic genes associated with periodontitis. The GeneCards database predicts the top 20 pathogenic genes in periodontal tissue. A box plot illustrates the expression patterns of these 20 genes in periodontitis and normal tissues, where yellow represents periodontal tissue, and blue represents normal tissue. Pearson’s correlation analysis shows the relationship between key genes and pathogenic genes. Red indicates a positive correlation, purple indicates a negative correlation. *P < 0.05; **P < 0.051; ***P < 0.001; ****P < 0.0001

We obtained pathogenic genes related to periodontitis from the GeneCards database (https://www.genecards.org/). We conducted an analysis of the expression levels of four key genes and the top 20 genes based on their Relevance score. The results revealed a significant correlation between the expression levels of the key genes and several genes associated with the disease (Fig. 6). Specifically, SLC2A14 and IL6 showed a significant positive correlation (Pearson *r* = 0.646), while SLC1A5 and TNFSF11 exhibited a significant negative correlation (Pearson *r* = -0.243).

### Performing Gene Set Enrichment Analysis (GSEA) to enrich functional pathways associated with key genes

**Fig. 7:**
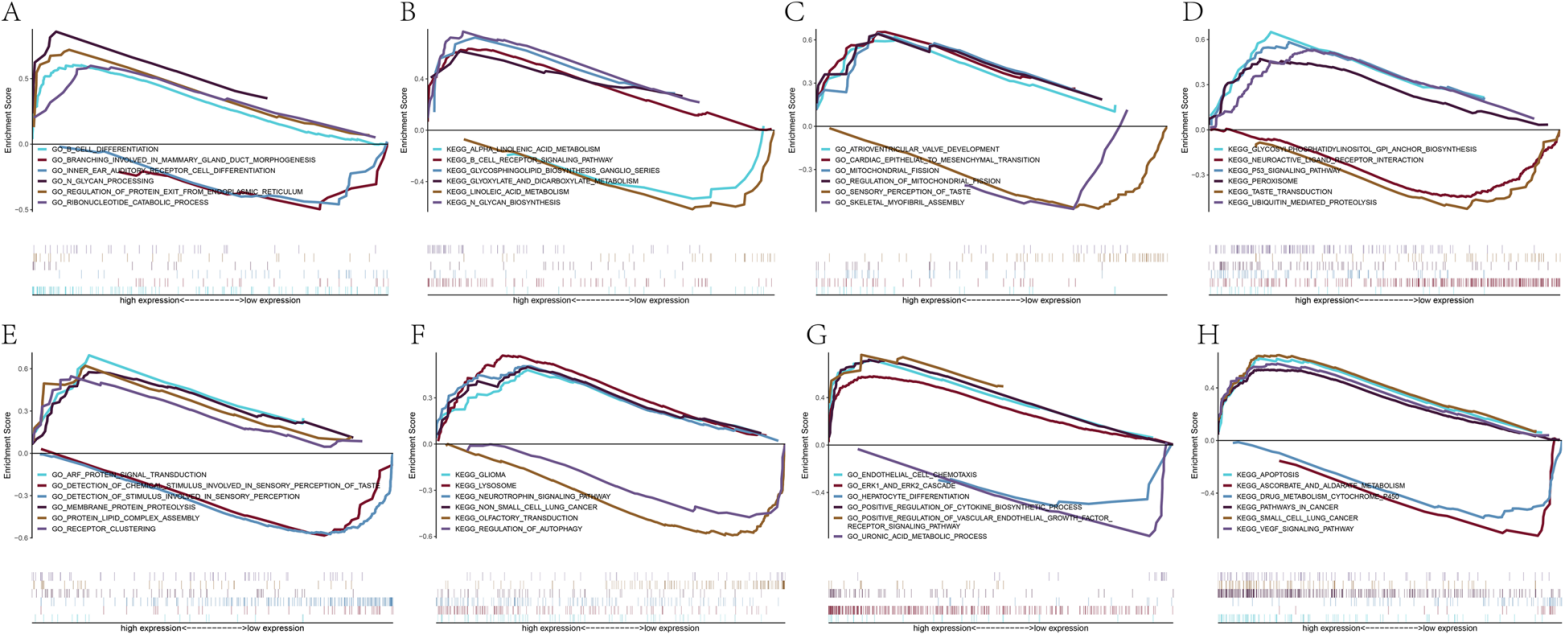
GSEA enrichment analysis. **A**-**H** The GO and KEGG enrichment plot for the top pathway in the 4 key genes including SLC1A5, SLC2A14, LURAP1L, and HERPUD1

In our subsequent research, we will investigate the specific signaling pathways enriched with four key genes, aiming to explore the potential molecular mechanisms through which these key genes impact the progression of periodontitis. We selected some highly significant pathways and showed them separately. The pathways enriched by SLC1A5 gene GO include the detection of chemical stimuli involved in sensor perception of taste and ARF PROTEIN SIGNAL TRANSDUCTION, and the pathways enriched by KEGG include GLIOMA and LYSOSOME. Pathways enriched by SLC2A14 gene GO include ERK1 AND ERK2 CASCADE, ENDOTHELIAL CELL CHEMOTAXIS, and those enriched by KEGG include ASCORBATE AND ALDARATE METABOLISM, APOPTOSIS, etc. The pathways enriched by LURAP1L gene GO include cardiac epithelial to mesochemical transition, atrio exotic valve development, etc. The pathways enriched by KEGG include glycosphindylinositol GPI anchor biosynthesis, neuroactive ligand-receptor interaction, and so on. Pathways enriched by HERPUD1 gene GO include branching involved in Mammary Gland duct morphogenesis and B CELL DIFFERENTIATION, while those enriched by KEGG include ALPHA LINOLENIC ACID METABOLISM and B CELL RECEPTOR SIGNALING PATHWAY (Fig. 7A-H). It is suggested that these signal pathways have a certain influence on the progress of the disease.

### The prediction of key genes’ transcriptional regulatory network and targeted drugs

**Fig. 8:**
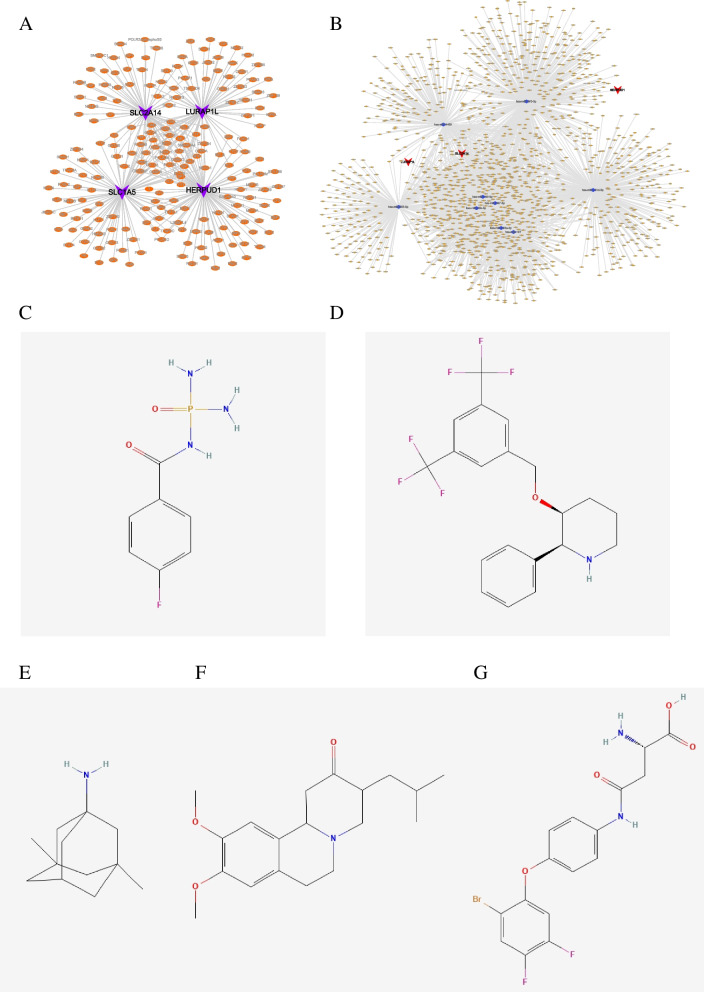
The prediction of key genes’ transcriptional regulatory network and targeted drugs. **A** The Cistrome DB online database shows the predicted transcription factors for key genes. **B** The miRWalk and ENCORI databases show the potential ceRNA net for four key genes. Dots represent gene or RNA names, and lines represent transcriptional regulatory relationships. **C**-**G** The Connectivity Map(CMap) revealed five potential targeted drugs flurofamide, L-733060, memantine, tetrabenazine, WAY-213613 for periodontitis. The diagram illustrates the chemical formulas of these five targeted drugs

We employed this set of four key genes for the gene set analysis in the current study, aiming to further investigate the transcriptional regulatory networks associated with these critical genes. Through the Cistrome DB online database, we predicted the associated transcription factors. Specifically, SLC1A5 was predicted to have 81 transcription factors, SLC2A14 was associated with 69 transcription factors, LURAP1L showed predictions for 69 transcription factors, and HERPUD1 was linked to 94 transcription factors. Finally, a transcriptional regulatory network related to periodontitis was visualized using Cytoscape (Fig. 8A). We predicted potential miRNAs and lncRNAs for four key genes using the miRWalk and ENCORI databases, respectively. Firstly, we extracted mRNA-miRNA pairs related to these four mRNAs from the miRWalk database, resulting in a total of 1,041 miRNAs. Subsequently, we retained only 54 mRNA-miRNA pairs detectable in either the TargetScan or miRDB databases, encompassing three mRNAs and nine miRNAs. Utilizing these nine miRNAs, we predicted interacting lncRNAs, identifying a total of 3,527 interaction pairs involving nine miRNAs and 1,497 lncRNAs. Finally, we constructed the ceRNA network using Cytoscape (v3.7), as illustrated in Fig. 8B. In the end, we categorized the differential mRNA into two groups: upregulated and downregulated. Using the Connectivity Map database for drug target prediction on differentially expressed genes, the results indicated a significant negative correlation between the expression profiles of flurofamide, L-733060, memantine, tetrabenazine, WAY-213613, and the disease perturbation profiles. These drugs demonstrated the ability to alleviate or even reverse the pathological state (Figs. 8C-G).

## Discuss

Although an increasing number of studies have gradually unveiled the role of ferroptosis, a novel and critical form of cell death, in the pathogenesis of periodontitis, the specific mechanisms through which it influences the onset and progression of this condition remain elusive [21–23]. Moreover, there is a scarcity of reports identifying key genes associated with ferroptosis that could serve as valuable clinical biomarkers for periodontitis. Therefore, in this study, we initially identified 36 candidate genes based on the differential expression of ferroptosis-related genes in normal versus periodontitis-affected samples. Subsequently, through protein–protein interaction networks and multi-dataset machine learning algorithms, we further narrowed down these candidates to four key ferroptosis-related genes differentially expressed in periodontitis, namely SLC1A5, SLC2A14, LURAP1L, and HERPUD1. We analyzed and validated the roles of these key genes in different subtypes of periodontitis through single-cell sequencing, focusing on their involvement in immune infiltration and GESA enrichment functions. This investigation further revealed the association of these ferroptosis-related key genes with molecular mechanisms involving immune infiltration and signal transduction in the disease progression of periodontitis. Ultimately, by predicting the ceRNA mechanisms or drug targets that may regulate these key genes through transcriptional regulatory networks, our study provides valuable and promising research directions for utilizing these key genes as diagnostic and therapeutic markers in the clinical management of periodontitis.

Programmed cell death encompasses apoptosis, necrosis, pyroptosis, and ferroptosis [24, 25]. The roles of apoptosis, necrosis, and pyroptosis-related genes in the pathogenesis of periodontitis have been extensively studied and reported in the literature [26–29]. In contrast, while some reports have shed light on the significance of ferroptosis in the pathogenicity of periodontitis, the specific genes and molecular mechanisms involved remain insufficiently understood, necessitating further experimental and bioinformatics research [30–32]. Therefore, this study primarily focuses on employing comprehensive bioinformatics analysis to identify ferroptosis-related genes in cells within human periodontal tissues affected by periodontitis. We conducted a bioinformatics analysis of the GSE16134 dataset from the GEO database, employing Random Forest and SVM machine learning algorithms to identify four differentially expressed ferroptosis-related key genes: SLC1A5, SLC2A14, LURAP1L, and HERPUD1.Subsequently, we explored the relationship of these four key genes with immune infiltration. Our findings indicate that the expression of HERPUD1 and SLC1A5 positively correlates with plasma cell infiltration and negatively with M1 macrophage infiltration. Conversely, the expression levels of LURAP1L and SLC2A14 show significant negative correlations with the infiltration of CD8 + T cells and naive CD4 + T cells. Further analysis of the associations of these key genes with immune-stimulatory, immune-suppressive, and chemotactic factors revealed a positive correlation between these four crucial ferroptosis-related genes and the level of immune infiltration. These comprehensive results indicate the significant role of these key genes in immune processes within the context of periodontitis.

Existing research indicates that SLC1A5 is an amino acid transporter, primarily responsible for the transport of alanine, serine, and cysteine. It plays a crucial role in maintaining normal cellular growth and the immune functions related to T lymphocyte activity [33, 34]; SLC2A14, a member of the glucose transporter family, is involved in glucose transport and metabolic regulation [35, 36]; LURAP1L influences the tumorigenicity of fibroblasts through NF-κB signaling [37]; HERPUD1, a protein associated with endoplasmic reticulum (ER) stress, is involved in the ER stress response and linked to the ubiquitin degradation system [38, 39]. It may also regulate cellular lipid responses by affecting lipid synthesis and metabolic pathways [40]. A common characteristic of these four key genes is their involvement in the ferroptosis process by influencing metabolic pathways related to amino acids, glucose, and lipid synthesis. Of particular interest is recent study have found that Bomidin, a novel recombinant antimicrobial peptide, acts as a ferroptosis inhibitor by activating the Keap1/Nrf2 signaling pathway. It has been demonstrated to regulate Keap1 protein stability by binding and ubiquitin–proteasome pathway, thereby alleviating inflammatory responses for the treatment of periodontitis [40], and this suggests that targeting ferroptosis-related genes for the treatment of periodontitis is feasible. Consequently, we reasonably speculate that these four key genes can serve as genetic adjuvant therapy to conventional treatments, impacting periodontitis clinical management by modulating immune functionality and metabolic reprogramming during the validation process, thereby improving clinical treatment through the ferroptosis pathway [41–43].

The ANN diagnostic model comprising four key genes—SLC1A5, SLC2A14, LURAP1L, and HERPUD1—demonstrated a high predictive efficacy for periodontitis with an AUC of 0.928 in the dataset. Periodontitis, a chronic inflammatory disease associated with bacterial biofilms, is characterized by the loss of periodontal support and has been traditionally regarded as a "silent disease," adversely affecting the oral health of over 700 million people globally. The specific pathogenesis of this multifactorial disease remains elusive. Our study links the pathogenesis of periodontitis with the ferroptosis pathway, building a bridge through the bioinformatics-predicted key genes SLC1A5, SLC2A14, LURAP1L, and HERPUD1.Existing research indicates that periodontal tissue in periodontitis exhibits dysregulation of iron-containing compounds such as ferritin, transferrin, and heme [44, 45]. Excess free iron catalyzes the Fenton reaction, leading to radical generation and systemic ROS influx. The periodontal pocket, characterized by an anoxic environment due to the predominance of subgingival anaerobic bacteria, is influenced by HIF, a primary regulator of many genes related to iron homeostasis and inflammation, including TFR1, erythropoietin, and ferritin [14]. This facilitates fatty acid deposition in the microenvironment [46]. The "microbial infection-ROS-inflammation" feed-forward mechanism appears to accelerate the body's susceptibility to infection, inflammation, and oxidative stress [47]. Studies have shown a close association between oxidative stress, lipid peroxidation, and periodontitis. Changes in GPX, the glutathione/oxidized glutathione ratio, myeloperoxidase (MPO) activity, lipid peroxides, and other markers in periodontitis patients indicate that an increase in GPX may be an antioxidative compensation in response to oxidative stress [48]. The striking similarities in marker alterations between periodontitis and iron-induced cell death suggest a potential correlation. Based on this, our study hypothesizes that ferroptosis-related genes, including SLC1A5, SLC2A14, LURAP1L, and HERPUD1, could be novel biomarkers for periodontitis. However, this study has limitations, including a small sample size and lack of clinical validation analysis. Therefore, further validation analyses on a larger sample scale are necessary.

## Conclusion

The ferroptosis-related genes SLC1A5, SLC2A14, LURAP1L, and HERPUD1 may serve as novel biomarkers for the diagnosis of periodontitis. These genes are likely to influence the progression of periodontitis by affecting immune infiltration and metabolic reprogramming through the ferroptosis pathway.

### Supplementary Information

Supplementary Material 1.

## Data Availability

The authors acquired periodontitis-related data for single-cell analysis by downloading the GSE171213 dataset from the NCBI GEO public database. The dataset comprises a total of nine samples, including five from diseased subjects and four from control subjects. The Series Matrix File data for GSE16134 was downloaded from the NCBI GEO public database, with the annotation file labeled as GPL570. The authors confirm that the data supporting the findings of this study are available within the article and its supplementary materials.
